# Characterization of Clade 7.2 H5 Avian Influenza Viruses That Continue To Circulate in Chickens in China

**DOI:** 10.1128/JVI.00855-16

**Published:** 2016-10-14

**Authors:** Liling Liu, Xianying Zeng, Pucheng Chen, Guohua Deng, Yanbing Li, Jianzhong Shi, Chunyang Gu, Huihui Kong, Yasuo Suzuki, Yongping Jiang, Guobin Tian, Hualan Chen

**Affiliations:** a State Key Laboratory of Veterinary Biotechnology, Harbin Veterinary Research Institute, Chinese Academy of Agricultural Sciences, Harbin, People's Republic of China; b College of Life and Health Sciences, Chubu University, Aichi, Japan; Wake Forest University

## Abstract

The H5N1 avian influenza viruses emerged in Southeast Asia in the late 20th century and have evolved into multiple phylogenetic clades based on their hemagglutinin (HA)-encoding genes. The clade 7.2 viruses were first detected in chickens in northern China in 2006, and vaccines specifically targeted to the clade were developed and have been used in poultry in China since 2006. During routine surveillance and disease diagnosis, we isolated seven H5 viruses between 2011 and 2014 that bear the clade 7.2 HA genes. Here, we performed extensive studies to understand how the clade 7.2 H5 viruses have evolved in chickens in China. Full genome sequence analysis revealed that the seven viruses formed two subtypes (four H5N1 viruses and three H5N2 viruses) and four genotypes by deriving genes from other influenza viruses. All of the viruses had antigenically drifted from the clade 7.2 viruses that were isolated in 2006. Pathogenicity studies of four viruses, one from each genotype, revealed that all of the viruses are highly pathogenic in chickens, but none of them could replicate in ducks. The four viruses exclusively bound to avian-type receptors and replicated only in the turbinates and/or lungs of mice; none of them were lethal to mice at a dosage of 10^6^ 50% egg infective doses (EID_50_). Our study indicates that although the clade 7.2 viruses have not been eradicated from poultry through vaccination, they have not become more dangerous to other animals (e.g., ducks and mice) and humans.

**IMPORTANCE** Animal influenza viruses can acquire the ability to infect and kill humans. The H5N1 viruses have been a concern in recent decades because of their clear pandemic potential. We sorted H5N1 influenza viruses into different phylogenetic clades based on their HA genes. The clade 7.2 viruses were detected in chickens in several provinces of northern China in 2006. Vaccines for these viruses were subsequently developed and have been used ever since to control infection of poultry. Here, we analyzed the genetic and biologic properties of seven clade 7.2 viruses that were isolated from chickens between 2011 and 2014. We found that after nearly 9 years of circulation in chickens, the clade 7.2 viruses still exclusively bind to avian-type receptors and are of low pathogenicity to mice, suggesting that these H5 viruses pose a low risk to human public health.

## INTRODUCTION

Influenza A viruses are categorized into different subtypes according to the antigenicity of their two surface glycoproteins, hemagglutinin (HA) and neuraminidase (NA). Currently, 16 different HA and 9 different NA subtypes of influenza viruses have been identified in avian species. Most avian influenza viruses are of low pathogenicity to poultry, although some viruses containing subtype H5 and H7 HAs are highly pathogenic for poultry and have caused numerous avian influenza outbreaks.

The H5N1 avian influenza viruses emerged in Southeast Asia in the later 20th century and have evolved into 10 phylogenetic clades (0 to 9) based on their HA-encoding genes (1). The viruses in a few clades continue to circulate in nature and to cause disease outbreaks in domestic poultry around the world. The clade 1 H5N1 viruses caused disease outbreaks in poultry and human infections and deaths in Viet Nam and Thailand in 2004; these viruses continue to circulate in poultry and reassort with other viruses, causing multiple disease outbreaks in Viet Nam (2–4). The clade 2 viruses have further evolved into different subclades and have infected and caused numerous disease outbreaks and deaths in wild birds, poultry, and humans (1, 5–8). Recently, multiple reassortant viruses containing the clade 2.3.4.4 HA and different subtypes of NA, including H5N2, H5N6, and H5N8, emerged and caused disease outbreaks in poultry in Asia, Europe, and North America (9–12). The clade 7 viruses were first detected in chickens in Xinjiang Province in China in 2005 (13). A subclade of these viruses, designated clade 7.2, emerged in chickens in Shanxi Province, China, in 2006 and then were widely detected in chickens in several provinces in northern China (13).

Vaccination is an important strategy to control H5 avian influenza viruses in poultry in several countries and regions (14–17). The vaccine strains used in China have been updated several times since 2004 to ensure an antigenic match between the vaccines and the prevalent strains (15). An inactivated vaccine containing the modified HA gene of the CK/SX/2/06 virus (the representative strain of clade 7.2), designated Re-4, was developed and has been used to control clade 7.2 virus infection of chickens in China since 2006 (16). A total of 41,325 billion doses of the H5N1 inactivated vaccine containing the Re-4 antigen were used in China from 2006 to 2012, which represents 60% of the total inactivated vaccines that were used in China and other countries during this period (15). Although disease outbreaks have been prevented, the viruses of the subclade were not eradicated. We isolated seven viruses that contain the HA of clade 7.2 from samples that we collected during routine disease surveillance and disease diagnosis between 2011 and 2014. Unlike the viruses of other clades, which mainly emerged and circulated in ducks and wild birds (5, 13, 18, 19), the clade 7.2 viruses have been mainly maintained in chickens, a species of poultry that is relatively well vaccinated in China. Therefore, analysis of these viruses will help us to understand how the H5N1 viruses have evolved in vaccinated bird flocks.

## MATERIALS AND METHODS

### Viruses.

The seven viruses were isolated in China between 2011 and 2014. Three viruses—A/chicken/Liaoning/S4092/2011(H5N1) (CK/LN/S4092/11), A/chicken/Shandong/S3/2014(H5N2) (CK/SD/S3/14), and A/chicken/Heilongjiang/S7/2014(H5N2) (CK/HLJ/S7/14)—were isolated from apparently healthy broilers in slaughterhouses during our routine surveillance, and four viruses, namely, A/chicken/Ningxia/2/2012(H5N1) (CK/NX/2/12), A/chicken/Gansu/5/2012(H5N1) (CK/GS/5/12), A/chicken/Gansu/6/2012(H5N1) (CK/GS/6/12), and A/chicken/Hebei/3/2013(H5N2) (CK/HeB/3/13), were isolated from samples that were sent to the our laboratory for diagnosis in suspected cases of avian influenza virus infection. All experiments with live H5 viruses were performed in a biosafety level 3 laboratory in the Harbin Veterinary Research Institute (HVRI), Chinese Academy of Agricultural Sciences (CAAS).

### Genetic and phylogenetic analyses.

Viral RNA was extracted from allantoic fluid by using the RNeasy minikit (Qiagen, Valencia, CA, USA) and reverse transcribed to cDNA. PCR amplification was performed by using segment-specific primers (sequences are available upon request), and the amplified products were purified and sequenced in a DNA analyzer (Applied Biosystems, Foster City, CA, USA). Sequence data were compiled by using the Seqman module of the DNAStar package, and phylogenetic analyses were carried out by using MEGA software (version 5.0) with a neighbor-joining algorithm. Bootstrap values were 1,000.

### Receptor-binding analysis.

Receptor specificity was analyzed by use of a solid-phase direct-binding assay as described previously (20–22) using two modified glycopolymers: α-2,3-siaylglycopolymer (Neu5Aca2-3Galb1-4GlcNAcb1-pAP [para-aminophenyl]-alpha-polyglutamic acid [α-PGA]) and α-2,6-sialylglycopolymer (Neu5Aca2-6Galb1-4GlcNAcb1-pAP–α-PGA). Chicken antiserum raised against the clade 7.2 virus CK/NX/2/12 was used as the primary antibody for the clade 7.2 viruses, and chicken antisera against A/duck/Guangxi/35/2001(H5N1) and A/Sichuan/1/2009(H1N1) were used as the primary antibodies for A/duck/Guangxi/35/2001(H5N1) and A/Sichuan/1/2009(H1N1) viruses, respectively. Horseradish peroxidase (HRP)-conjugated goat-anti-chicken IgG (Sigma-Aldrich, St. Louis, MO, USA) was used as the secondary antibody. Absorbance was determined at 490 nm, and the cutoff value was defined as the background absorbance of the well containing 100 ng of glycopolymer without the virus.

### Animal experiments.

All animal studies were conducted in accordance with the recommendations of the Guide for the Care and Use of Laboratory Animals of the Ministry of Science and Technology of China, and the protocols were reviewed and approved by the Committee on the Ethics of Animal Experiments of the HVRI, CAAS.

To assess the pathogenicity of virus isolates, the intravenous pathogenicity index (IVPI) was determined according to the recommendation of the Office International des Epizooties (OIE). Specific-pathogen-free (SPF) 6-week-old White Leghorn chickens (*n* = 10 per group) housed in isolator cages were inoculated intravenously (i.v.) with 0.1 ml of virus-containing bacteria-free allantoic fluid diluted 1:10 (dosage range, 6.2 to 6.5 log_10_ 50% egg infective doses [EID_50_]) and were observed for 10 days for signs of disease and death.

Four-week-old SPF Sheldrake ducks (*n* = 13 per group) were intranasally (i.n.) inoculated with 10^6^ EID_50_ of H5N1 or H5N2 viruses in a volume of 0.1 ml, 3 ducks from each group were killed on day 3 postinoculation (p.i.), and their organs were collected for virus titration. Swabs from the remaining 10 ducks were collected on days 3, 5, and 7 for the detection of virus shedding, and sera were collected at 2 weeks p.i. to test the HA-inhibitory (HI) antibody against H5N1 virus.

Eight 6-week-old female BALB/c mice (Beijing Vital River Laboratories, Beijing, China) were lightly anesthetized with dry ice and inoculated i.n. with 10^6^ EID_50_ of each H5 virus in 50 μl of phosphate-buffered saline (PBS). Three mice from each group were sacrificed by use of CO_2_ on day 3 postinfection to determine virus titers in the turbinate, lungs, kidneys, spleen, and brain; the remaining five mice were monitored daily for disease signs and mortality.

### Antigenic analyses.

Antigenic analysis was performed by using the HI assay with 0.5% chicken erythrocytes (23) and chicken antisera generated in 6-week-old SPF chickens inoculated with 0.5 ml of inactivated oil-emulsified vaccine.

### Vaccination and challenge study in chickens.

The H5N1 inactivated vaccine Re-4 was produced from a genetic reassortant virus that contains modified HA and NA genes of the clade 7.2 virus CK/SX/2/06 and six internal genes of the A/Puerto Rico/8/34 (PR8) virus; this vaccine virus has been used in chickens in China since 2006 (16). The H5N1 inactivated vaccine Re-7 was produced from a genetic reassortant virus that contains modified HA and NA genes of the clade 7.2 virus CK/LN/S4092/11 and six internal genes of the PR8 virus. These vaccines were supplied by the Harbin Weike Biotechnology Development Company (Harbin, China). Two groups of 30 3-week-old SPF chickens were intramuscularly injected with 0.3 ml of the Re-4 and Re-7 vaccines containing about 2.8 μg of HA, respectively. Fifteen similar chickens were injected with PBS as a control. Three weeks postvaccination (p.v.), 10 chickens from each vaccinated group and five chickens from the control group were challenged i.n. with 10^5^ EID_50_ of the test virus. Prechallenge sera were collected from all of the chickens and tested for HI antibody titers against the vaccine strain and the challenge virus. Oropharyngeal and cloacal swabs from all live chickens were collected on days 3 and 5 postchallenge (p.c.) to determine virus titers in eggs, and the chickens were observed for disease symptoms and death for 2 weeks.

### Accession number(s).

The genome sequences of the seven viruses analyzed in this study are available in GenBank (accession numbers KX160148 to KX160203).

## RESULTS

### Molecular and phylogenetic analyses.

**FIG 1:**
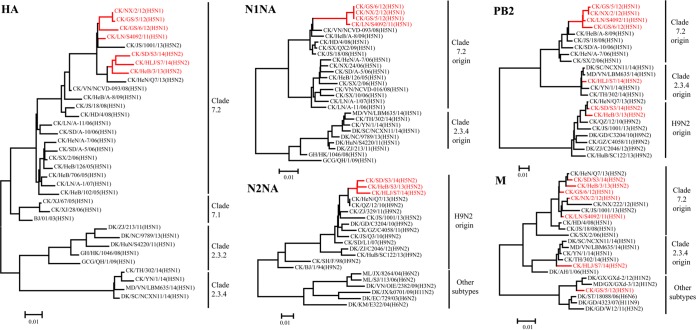
Phylogenetic analyses of the clade 7.2 H5 viruses isolated in China between 2011 and 2014. The phylogenetic trees were generated by using the neighbor-joining method and the MEGA 5.0 software package. The regions of nucleotide sequence used for the phylogenetic analyses were as follows: HA, 29 to 1732; N1NA, 20 to 1420; N2NA, 1 to 1458; PB2, 28 to 2289; and M, 26 to 1007. Viruses obtained in this study are shown in red. AH, Anhui; BJ, Beijing; CK, chicken; DK, duck; EC, eastern China; GCG, great crested grebe; GD, Guangdong; GH, gray heron; GS, Gansu; GX, Guangxi; GZ, Guizhou; HD, Huadong; HeB, Hebei; HeN, Henan; HK, Hong Kong; HLJ, Heilongjiang; HuB, Hubei; HuN, Hunan; JS, Jiangsu; JX, Jiangxi; KM, Kingmen; LN, Liaoning; MD, mallard; ML, magpie robin; NC, Nanchang; NX, Ningxia; QH, Qinghai; QZ, Qianzhou; SC, Sichuan; SD, Shandong; SH, Shanghai; SJ, Sanjiang; ST, Shantou; SX, Shanxi; TH, Tonghai; VN, Vietnam; XJ, Xinjiang; YN, Yunnan; ZJ, Zhejiang.

We fully sequenced the genomes of all seven viruses. The HA genes of these viruses shared 96.5% to 99.7% identity at the nucleotide level and clustered together with the previously reported clade 7.2 viruses in the phylogenetic tree (Fig. 1). All seven isolates had a polybasic motif (-RRRKR-) at the HA cleavage site. The NA genes of the four H5N1 viruses shared 99.5% to 100% identity at the nucleotide level and clustered with other previously reported clade 7.2 viruses in the phylogenetic tree (Fig. 1). The NA genes of the three H5N2 viruses shared 98.2% to 98.6% identity at the nucleotide level and clustered with H9N2 viruses that were detected in China in recent years (Fig. 1).

The six internal genes of the seven viruses showed distinct diversity, with the basic polymerase 2 (PB2), PB1, acidic polymerase (PA), nucleoprotein (NP), matrix (M), and nonstructural protein (NS) genes of the seven viruses sharing 83.7% to 99.9%, 88.1% to 100%, 85.8% to 100%, 92.3% to 99.7%, 91.2% to 99.5%, and 84.8% to 100% identity, respectively, at the nucleotide level. The PB2, PB1, PA, NP, and NS genes of these viruses had similar topologies in the phylogenetic trees (Fig. 1, represented by PB2) and clustered in the clade 7.2 virus branch, the clade 2.3.4 virus branch, and the H9N2 virus branch. The M genes of five viruses and one virus clustered in the clade 7.2 virus branch and the clade 2.3.4 virus branch, respectively, whereas the M gene of another virus clustered with the M genes of other virus subtypes that were detected in ducks or wild birds (Fig. 1).

**FIG 2:**
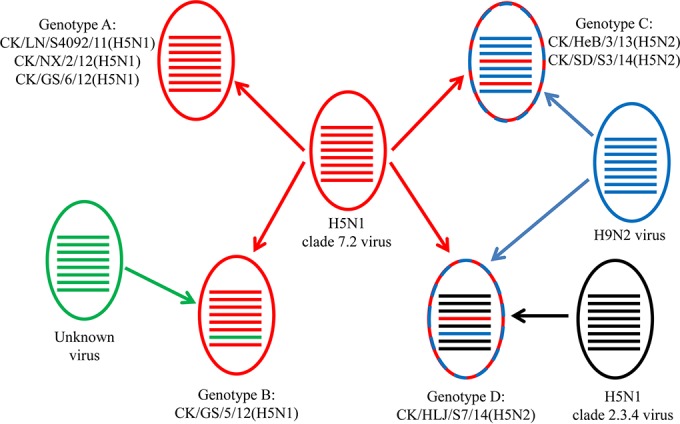
Genotypes of the clade 7.2 H5 avian influenza viruses. The eight bars represent the eight gene segments of influenza virus: from top to bottom, PB2, PB1, PA, HA, NP, NA, M, and NS.

The seven viruses formed four genotypes (Fig. 2). The three viruses in genotype A retained the entire eight gene segments of the early clade 7.2 virus, and the CK/GS/5/12(H5N1) virus in genotype B is a reassortant bearing seven genes of a clade 7.2 virus and the M gene of an unknown virus. The two H5N2 viruses in genotype C are reassortants bearing the HA and M genes of clade 7.2 viruses and the other six genes of H9N2 viruses. The H5N2 virus in genotype D is a triple reassortant that derived its HA, NA, and six other genes from a clade 7.2 H5 virus, an H9N2 virus, and a clade 2.3.4 H5 virus, respectively (Fig. 2).

### Antigenic analysis.

Influenza virus easily undergoes antigenic drift during circulation in nature. To understand the antigenic properties of these clade 7.2 viruses, we performed HI assays by using antisera to different H5 viruses generated in SPF chickens. The antiserum against DK/AH/1/06 virus (clade 2.3.4) cross-reacted with the early clade 7.2 virus CK/SX/2/06 with an HI titer only 4-fold lower than the homologous titer but reacted with the clade 7.2 viruses isolated between 2011 and 2014 with HI titers 32- to 256-fold lower than the homologous titers (Table 1). The antiserum against DK/GD/S1322/10 virus (clade 2.3.2) cross-reacted poorly with all of the clade 7.2 viruses, with HI titers 8- to 64-fold lower than the homologous titers (Table 1). The antiserum against the early clade 7.2 virus CK/SX/2/06 cross-reacted poorly with the recent clade 7.2 viruses, with HI titers 16- to 128-fold lower than the homologous titers (Table 1). The antiserum against the recent clade 7.2 virus CK/LN/S4092/11 cross-reacted poorly with the DK/AH/1/06, DK/GD/S1322/10, and CK/SX/2/06 viruses but well with all the recent clade 7.2 viruses (Table 1). These results indicate that the recent clade 7.2 viruses have antigenically drifted from other H5 viruses detected in China.

**TABLE 1:** Cross-reactive HI antibody titers of serum induced by different H5N1 vaccines against recent clade 7.2 viruses

Virus	Clade	Isolation date of the recent clade 7.2 virus	Antibody titer of antiserum induced by vaccine (HA gene donor virus)^*a*^			
			Re-5 (DK/AH/1/06)	Re-6 (DK/GD/S1322/10)	Re-4 (CK/SX/2/06)	Re-7 (CK/LN/S4092/11)
DK/AH/1/06(H5N1)	2.3.4	NA^*b*^	**512**	8	64	8
DK/GD/S1322/10(H5N1)	2.3.2	NA	16	**256**	32	4
CK/SX/2/06(H5N1)	7.2	NA	128	8	**1,024**	16
CK/LN/S4092/11(H5N1)	7.2	8 November 2011	8	16	64	**512**
CK/NX/2/12(H5N1)	7.2	18 April 2012	16	32	64	512
CK/GS/5/12(H5N1)	7.2	5 June 2012	16	16	64	256
CK/GS/6/12(H5N1)	7.2	8 June 2012	16	16	64	256
CK/HeB/3/13(H5N2)	7.2	20 December 2013	2	4	8	256
CK/SD/S3/14(H5N2)	7.2	17 January 2014	2	4	8	256
CK/HLJ/S7/14(H5N2)	7.2	14 August 2014	2	4	8	256

a Antisera were generated by vaccinating SPF chickens with oil-emulsified inactivated vaccines as indicated. Homologous titers are shown in boldface.

b NA, not applicable.

**FIG 3:**
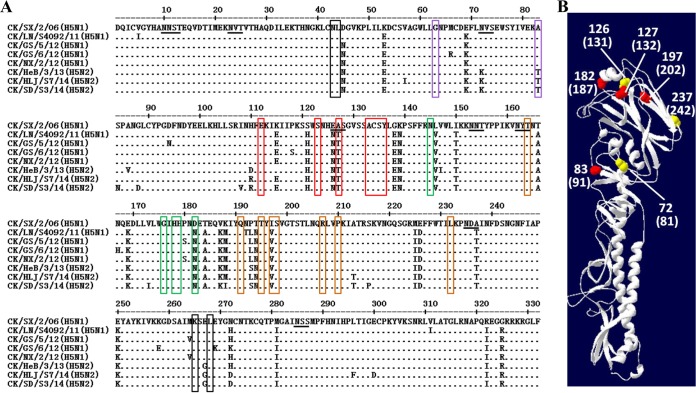
Key amino acid changes in the HA proteins of the clade 7.2 H5 avian influenza viruses. (A) Alignment of HA1 protein sequences. Antigenic sites A, B, C, D, and E are boxed in red, green, black, yellow, and purple, respectively. The potential glycosylation sites are underlined. (B) HA monomer of CK/SX/2/06 virus. The amino acid substitutions in antigenic sites of other clade 7.2 viruses are shown in red, and the glycosylation site changes are shown in yellow. The corresponding H3 numbering is shown in parentheses.

Detailed analysis revealed many amino acid changes in the HA1 of the recent clade 7.2 viruses compared with that of the CK/SX/2/06 virus, and some of these changes occurred in antigenic sites A, B, D, and E (Fig. 3). Three amino acid changes resulted in the presence of two potential glycosylation sites in the HA of the recent clade 7.2 strains (Fig. 3). Which changes contributed to these observed antigenic differences requires further investigation.

### Receptor-binding specificity.

**FIG 4:**
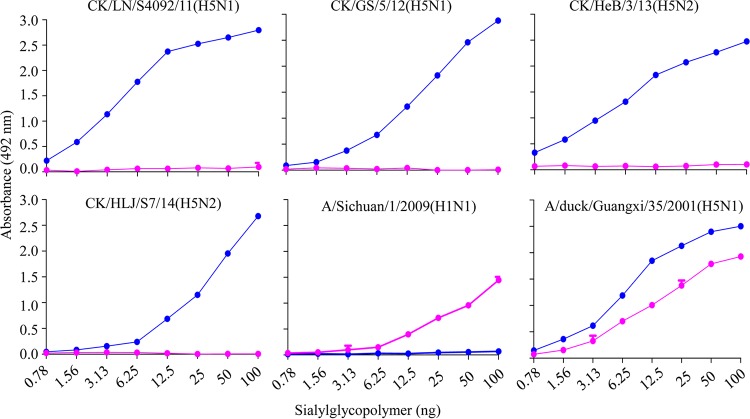
Characterization of the receptor-binding properties of H5 clade 7.2 viruses. The binding of the viruses to biotinylated α-2,3 (blue) and α-2,6 (pink) sialylglycans was tested. A/Sichuan/1/2009(H1N1) and A/duck/Guangxi/35/2001(H5N1) were used as controls. The data are expressed as means and standard deviations (SD) of three repeats.

The receptor-binding preference of the HA plays an important role in the transmission of influenza viruses in humans. The highly transmissible human influenza viruses exclusively bind to α-2,6-linked sialic acids (Sias) (human-type receptors) (24–27). Pappas et al. reported that the early H2N2 virus that mainly bound to the α-2,3-linked sialic acids was not transmitted in ferrets, whereas the highly transmissible H2N2 virus bound to α-2,6-linked sialic acids with higher affinity than to α-2,3-linked sialic acids (25). Some naturally isolated H5N1 viruses have acquired mutations that resulted in loss of the glycosylation site at positions 158 to 160 (H3 numbering) in HA, which increased their ability to bind to human-type receptors (28–30), and several studies using laboratory-adapted strains or mutants have identified combinations of amino acid changes, such as 158D/224K/226L, 196R/226L/228S, or 110Y/160A/226L/228S, in the HA protein that allow H5N1 viruses to recognize human-type receptors (31–33). Although none of these amino acid changes were detected in the clade 7.2 viruses, we investigated the receptor-binding specificities of four clade 7.2 viruses, one from each genotype, by using a solid-phase binding assay, as described previously (20–22). We found that all of these clade 7.2 viruses exclusively bound to α-2,3-glycan, whereas the human 2009 pandemic H1N1 virus A/Sichuan/1/09 exclusively bound to α-2,6-glycan and the H5N1 virus A/duck/Guangxi/35/2001 virus bound to both glycans (Fig. 4).

### Studies in chickens, ducks, and mice.

Although the motif of multiple basic amino acids in the HA cleavage site is a prerequisite for H5 and H7 viruses to be highly lethal in chickens, previous studies have shown that some H5 viruses bearing the motif are nonpathogenic for chickens (34–36). Four of the seven viruses we isolated came from apparently healthy broilers during our routine surveillance. Therefore, to investigate the virulence of these recent clade 7.2 viruses, we tested the IVPIs of four selected viruses, one from each genotype. Chickens inoculated with any of the four test viruses showed disease symptoms and died during the 10-day observation period, yielding IVPIs of 2.84 to 2.97 (Table 2). These results show that all of these viruses are highly pathogenic in chickens. The broilers that were carrying these viruses but did not show any signs of disease may have been protected by vaccination or by maternal antibodies that they acquired from vaccinated breeder hens.

**TABLE 2:** Replication and virulence of clade 7.2 H5 viruses in chickens, ducks, and mice

Virus (genotype)	EID_50_ of the viral stock (log_10_/ml)	IVPI in chickens	Ducks		Mice		
			No. shedding virus/surviving/total	No. seroconverting/total	Replication^*a*^		% survival
					Turbinate	Lung	
CK/LN/S4092/11(H5N1) (A)	8.2	2.97	0/10/10	5/10	1/3 (1.5)	3/3 (2.5)	100
CK/GS/5/12(H5N1) (B)	8.5	2.91	0/10/10	0/10	3/3 (3.5)	3/3 (3.7)	100
CK/HeB/3/13(H5N2) (C)	8.4	2.95	0/10/10	4/10	0/3	2/3 (2.0)	100
CK/HLJ/S7/14(H5N2) (D)	8.2	2.84	0/10/10	2/10	1/3 (0.8)	1/3 (1.2)	100

a No. positive/total (mean titer [log_10_EID_50_/ml]).

We previously reported that the early clade 7.2 viruses cannot replicate in ducks and have low pathogenicity in mice (13). Some of the recent clade 7.2 viruses derived certain genes from viruses of other clades that were circulating mainly in ducks and wild birds or from H9N2 viruses. We therefore investigated whether these genetic changes altered the replication properties of the viruses in ducks and mice.

Groups of 13 SPF ducks were i.n. inoculated with 10^6^ EID_50_ of test virus, and 3 of them were killed on day 3 p.i. in order to check viral titers in organs, including brains, lungs, spleens, and kidneys. Oropharyngeal and cloacal swabs were collected from the other 10 ducks on days 3 and 5 p.i. and titrated in eggs. However, virus replication was not detected in any ducks inoculated with any of the four test viruses (data not shown), and no virus shedding, disease signs, or deaths were noted in any ducks during the 2-week observation period, indicating that the viruses were unable to replicate in ducks. Five, four, and two ducks in the CK/LN/S4092/11-, CK/HeB/3/13-, and CK/HLJ/S7/14-inoculated groups, respectively, seroconverted by the end of the observation period (Table 2), which may have been due to direct antigen exposure rather than as a result of virus replication.

BALB/c mice have been widely used as a mammalian model to evaluate the virulence of influenza virus, and previous studies have shown that H5N1 virus virulence in mice correlates with its virulence in humans (37, 38). In the present study, eight 6-week-old female BALB/c mice were i.n. inoculated with 10^6^ EID_50_ of test H5 virus. Three mice from each group were sacrificed on day 3 p.i. to determine virus titers in the turbinate, lungs, kidneys, spleen, and brain, and the remaining five mice were monitored daily for disease signs and mortality for 2 weeks. The replication of CK/LN/S4092/11 virus was detected in the turbinates of one mouse, with a titer of 1.5 log_10_ EID_50_, and in the lung tissues of all three mice, with a mean titer of 2.5 log_10_ EID_50_ (Table 2). The CK/GS/5/12 virus was detected in all three mice in the lungs and turbinates with mean titers of 3.7 log_10_ EID_50_ and 3.5 log_10_ EID_50_, respectively (Table 2). CK/HeB/3/13 replication was detected in the lungs of two of three mice with a mean titer of 2.0 log_10_ EID_50_ but was not detected in the turbinates of any mice (Table 2). The replication of CK/HLJ/S7/14 was detected in one mouse with titers of 0.8 log_10_ EID_50_ and 1.2 log_10_ EID_50_ in the turbinate and lung, respectively. No virus was detected in the spleen, kidney, or brain of any mice. None of the viruses killed any mice during the observation period (Table 2). These results indicate that the recent clade 7.2 viruses are of low pathogenicity in mice.

### Protective efficacy of the H5N1 vaccines used in China.

The Re-4 inactivated vaccine, which was produced from a seed virus containing the modified HA and NA genes of CK/SX/2/06 and the internal genes of PR8, has been used in northern China against clade 7.2 H5 viruses since 2006. After the antigenically drifted clade 7.2 virus CK/LN/S4092/11 was detected during routine surveillance, we developed a new vaccine, Re-7, with a seed virus that bears the modified HA and NA genes of CK/LN/S4092/11 and the internal genes of PR8 by using the same strategy described previously (39). In this study, we compared the protective efficacies of the two vaccines in chickens against challenges with three clade 7.2 viruses: CK/LN/S4092/11 (the Re-7 vaccine strain's HA and NA donor virus), CK/GS/5/12(H5N1), and CK/HeB/3/13(H5N2).

As shown in Table 3, 3 weeks after a single inoculation with the Re-4 vaccine, the mean HI antibody titers in the three groups ranged from 7.4 to 7.9 log_2_ against the vaccine strain and from 2.9 to 5.1 log_2_ against the challenge viruses. Among the Re-7-vaccinated chickens, the mean HI antibody titers in the three groups ranged from 7.7 to 8.1 log_2_ against the vaccine strain and from 6.2 to 7.2 log_2_ against the challenge viruses (Table 3). HI antibody was not detectable in the control chickens.

**TABLE 3:** Protective efficacies of Re-4 and Re-7 vaccines against H5 clade 7.2 viruses in chickens

Challenge virus	Vaccine	Group	Mean HI antibody titers against vaccine strain/challenge virus (log_2_)^*a*^	No. shedding/total (log_10_ EID_50_)^*b*^				No. surviving/total
				Day 3 p.c.		Day 5 p.c.		
				Oropharyngeal	Cloacal	Oropharyngeal	Cloacal	
CK/LN/S4092/11(H5N1)	Re-4	Vaccinated	7.4/4.6	0/10	0/10	0/10	0/10	10/10
	Re-7	Vaccinated	7.7/7.1	0/10	0/10	0/10	0/10	10/10
	None	Control	<2	5/5 (3.5 ± 0.6)	5/5 (2.7 ± 0.3)	5/5 (3.3 ± 0.8)	5/5 (3.7 ± 0.4)	0/5
CK/GS/5/12(H5N1)	Re-4	Vaccinated	7.9/5.1	0/10	0/10	0/10	0/10	10/10
	Re-7	Vaccinated	7.8/6.2	0/10	0/10	0/10	0/10	10/10
	None	Control	<2	5/5 (2.6 ± 0.5)	5/5 (2.4 ± 0.1)	4/4 (3.1 ± 0.6)	3/4 (3.2 ± 0.4)	0/5
CK/HeB/3/13(H5N2)	Re-4	Vaccinated	7.7/2.9	2/10 (2.9 ± 0.5)	2/10 (2.1 ± 0.5)	8/10 (2.3 ± 0.4)	5/10 (2.1 ± 0.7)	5/10
	Re-7	Vaccinated	8.1/7.2	0/10	0/10	0/10	0/10	10/10
	None	Control	<2	5/5 (2.7 ± 0.7)	2/5 (2.6 ± 0.1)	4/5 (2.4 ± 0.3)	2/5 (2.5)	0/5

a HI antibody titers were measured by using the vaccine antigen with antisera collected 3 weeks after vaccination.

b Oropharyngeal and cloacal swabs were collected on days 3 and 5 after viral challenge and titrated in SPF eggs. The titers shown are the means ± standard deviations (SD) calculated for virus-shedding chickens.

Chickens immunized with the Re-4 vaccine were completely protected from challenge with CK/LN/S4092/11 and CK/GS/5/12; however, virus shedding was detected in 8 of 10 chickens challenged with the CK/HeB/3/13 virus, and 5 of the chickens died during the observation period. Chickens immunized with the Re-7 vaccine were completely protected from challenges with all three viruses. No virus shedding, clinical signs, or deaths were observed. All of the control chickens shed viruses and died during the observation period. These results indicate that the Re-7 vaccine is better than the Re-4 vaccine at preventing infection with the recent clade 7.2 viruses.

## DISCUSSION

Here, we conducted a detailed analysis of the genetic variation and biological properties of recent clade 7.2 viruses. We found that the viruses derived gene segments from viruses of other clades or subtypes, formed three new genotypes, were highly pathogenic in chickens, could not replicate in ducks, retained their avian-type receptor-binding properties, and were of low pathogenicity in mice. Our results indicate that although the recent clade 7.2 viruses have antigenically drifted from the clade 7.2 viruses isolated in 2006, their host range and virulence in mammals have not changed markedly.

The H9N2 influenza viruses circulate widely in domestic poultry across many countries (40–44). The H9N2 viruses isolated in China form multiple genotypes (21, 41) and were the source of the internal genes of the novel H7N9 viruses and the H10N8 viruses that have infected humans in China (22, 45, 46). In the current study, we found that the genotype C virus and genotype D virus derived six gene segments (PB2, PB1, PA, NP, NA, and NS) and one gene segment (NA), respectively, from H9N2 virus, further demonstrating that cocirculation of different viruses facilitates the generation of influenza virus reassortants.

Wild birds and ducks are regarded as the natural reservoirs of influenza viruses. Although several important clades of H5N1 viruses were originally detected in ducks or wild birds (18, 47–50), we do not know if the ancestor of clade 7.2 was also maintained in wild birds. The clade 7.2 virus was first detected in chickens as a reassortant that derived its genes from different sources and lacked the ability to replicate in ducks (13). Our present study showed that the clade 7.2 viruses are still unable to replicate in ducks, even though some strains have derived genes from other duck viruses. This explains why clade 7.2 viruses have never been isolated from ducks since their emergence in 2006. The clade 7.2 viruses are therefore a useful model to investigate the underlying mechanism for host range restriction of influenza viruses.

H5N1 avian influenza viruses have not only caused disease outbreaks in poultry and wild birds but have also infected hundreds of humans. In our previous study of viruses isolated from ducks in Southern China between 1999 and 2002, we observed an increasing level of pathogenicity in mice with the progression of time (51). Li et al. compared the replication and virulence of H5N1 viruses from different clades and found that most clade 2.3.4 viruses and all clade 2.2 viruses were highly lethal in mice but that the replication of the clade 7 viruses was restricted to the respiratory system of mice and that all of the viruses were of low pathogenicity in mice (13). It is interesting that the present study indicates that the pathogenicity of the clade 7.2 viruses for mammals has not increased despite their circulation for several years in chickens.

Binding to human-type receptors is important for avian influenza viruses to infect and be transmitted in humans. Several studies have shown that many subtypes of avian influenza viruses are able to bind to human-type receptors, even though they retain a high affinity for avian-type receptors (20–22, 45). Li et al. reported that recent H9N2 avian influenza virus isolates exclusively bind to human-type receptors and that some are transmissible in ferrets (21). Some H5N1 avian influenza viruses isolated from ducks and wild birds have acquired the ability to bind to human-type receptors (30), and loss of the glycosylation site at positions 158 to 160 of HA (H3 numbering) contributes to this property (28). Our present study showed that the clade 7.2 viruses exclusively bind to avian-type receptors and therefore carry a low risk of transmission to humans.

Vaccination has long been a major strategy for H5 avian influenza control in China. However, because of differences in the implementation of this strategy, its effectiveness varies depending on the region and the bird species. For example, in Hong Kong, even though the lethal H5N1 viruses have always been detected in wild birds in the region (47, 48), the viruses have not been isolated from poultry since the compulsive vaccination strategy was strictly implemented in 2002. In mainland China, however, it is impossible to vaccinate every single bird because of the huge volume and types of species of poultry reared. Our surveillance data show that less than 30% of ducks and broilers and about 70% of chicken layers in mainland China have been inoculated with the H5 vaccine. Therefore, when a virus is introduced into poultry populations, it may be able to infect the birds that do not have enough immunity and to circulate for some time. Our present study indicates that even though the low vaccination coverage did not completely prevent virus infection in poultry, circulation among vaccinated flocks did not drive the H5 viruses to become more dangerous to other animals (e.g., ducks and mice) or to humans.
